# Dissimilatory nitrate reductions in soil *Neobacillus* and *Bacillus* strains under aerobic condition

**DOI:** 10.71150/jm.2411019

**Published:** 2025-02-27

**Authors:** Seohyun Ahn, Min Cho, Michael J. Sadowsky, Jeonghwan Jang

**Affiliations:** 1 Division of Biotechnology and Advanced Institute of Environment and Bioscience, Jeonbuk National University, Jeonbuk 54596, Republic of Korea; 2 BioTechnology Institute, Department of Soil, Water & Climate, and Department of Microbial and Plant Biology, University of Minnesota, Minnesota 55108, USA

**Keywords:** soil bacilli, simultaneous nitrate conversions, aerobic dissimilatory nitrate reductions, denitrification, dissimilatory nitrate reduction to ammonium

## Abstract

Denitrification and dissimilatory nitrate reduction to ammonium (DNRA) were thought to be carried-out by anaerobic bacteria constrained to anoxic conditions as they use nitrate (NO_3_^-^) as a terminal electron acceptor instead of molecular O_2_. Three soil bacilli, *Neobacillus* spp. strains PS2-9 and PS3-12 and *Bacillus salipaludis* PS3-36, were isolated from rice paddy field soil in Korea. The bacterial strains were selected as possible candidates performing aerobic denitrification and DNRA as they were observed to reduce NO_3_^-^ and produce extracellular NH_4_^+^ regardless of oxygen presence at the initial screening. Whole genome sequencing revealed that these strains possessed all the denitrification and DNRA functional genes in their genomes, including the *nirK*, *nosZ*, *nirB*, and *nrfA* genes, which were simultaneously cotranscribed under aerobic condition. The ratio between the assimilatory and dissimilatory NO_3_^-^ reduction pathways depended on the availability of a nitrogen source for cell growth, other than NO_3_^-^. Based on the phenotypic and transcriptional analyses of the NO_3_^-^ reductions, all three of the facultative anaerobic strains reduced NO_3_^-^ likely in both assimilatory and dissimilatory pathways under both aerobic and anoxic conditions. To our knowledge, this is the first report that describes coexistence of NO_3_^-^ assimilation, denitrification, and DNRA in a *Bacillus* or *Neobacillus* strain under aerobic condition. These strains may play a pivotal role in the soil nitrogen cycle.

## Introduction

Nitrate (NO_3_^-^) is the most mobile nitrogen (N) form and due to its great solubility it is considered to be the main N pollutant leached from soil (Kundu & Mandal, 2009; Lee et al., 2023). The NO_3_^-^ in environments can be reduced by microbes in assimilatory and dissimilatory pathways (Sparacino-Watkins et al., 2014). In the assimilatory pathway, NO_3_^-^ is taken into a microbial cell and reduced to NO_2_^-^ by assimilatory nitrate reductase in the cytoplasm (Nas) or extracellular NO_2_^-^ produced by dissimilatory nitrate reductase in the periplasm (Nap) or cytoplasm (Nar) could be taken back into the cells (Goddard et al., 2017; Stolz & Basu, 2002). Subsequently, it is said that the produced NO_2_^-^ is converted to NH_4_^+^ by nitrite reductase (NasB or NirB) followed by integration into N-containing organic molecules (Huang et al., 2022; Kuypers et al., 2018; Yılmaz et al., 2022), but the regulation and molecular mechanism of NirB is still unclear. In contrast, in the dissimilatory pathway, NO_3_^-^ can be reduced to NH_4_^+^ or dinitrogen gas (N_2_) via dissimilatory nitrate reduction to ammonium (DNRA) or denitrification, respectively. The NH_4_^+^ produced by cytoplasmic nitrite reductase (NirB) or periplasmic nitrite reductase (NrfA) is released from the microbial cell during the DNRA process (Pandey et al., 2020; Welsh et al., 2014). In contrast, complete denitrification is comprised of a series of sequential enzymatic reactions reducing NO_3_^-^ to nitrite (NO_2_^-^) by nitrate reductase (Nap or Nar), NO_2_^-^ to nitric oxide (NO) by nitrite reductase (NirK or NirS), NO to nitrous oxide (N_2_O) by nitric oxide reductase (Nor), and finally N_2_O to N_2_ by nitrous oxide reductase (Nos) (Zumft, 1997).

Denitrification is thought to be the main NO_3_^-^-removing microbial process in environments as NO_3_^-^ is reduced to gaseous N forms such as N_2_O and N_2_. It contributes to soil N loss and soil N is often the limiting nutrient for crop growth (Philippot et al., 2007; Shan et al., 2016). Moreover, since the process can effectively remove excess NO_3_^-^ produced from nitrification process of wastewater treatment, many studies have been done to examine factors affecting optimal NO_3_^-^ removal efficiency and minimum N_2_O production in wastewater-treating bioreactors (Gan et al., 2023; Lee et al., 2023; Nordström et al., 2021; Schipper et al., 2010). Unlike denitrification, DNRA would help soil N conservation by decreasing N losses through NO_3_^-^ leaching and N_2_O emission by converting NO_3_^-^ to cationic NH_4_^+^ which is easily adsorbed by soil particles (Giblin et al., 2013; McNeill & Unkovich, 2007; Zhang et al., 2015). Denitrification and DNRA compete for the substrate NO_2_^-^ and the dominance of one pathway over the other is regulated by multiple factors in the environment (Pandey et al., 2020; Yoon et al., 2015). For examples, Heo et al. (2020) reported that a high C/N ratio and low nitrate concentration favors DNRA over denitrification (Heo et al., 2020). In addition, bacteria with the capacity to simultaneously carry-out heterotrophic nitrification and aerobic denitrification (HNAD) have also been reported even though the mechanisms of HNAD are still unclear and under debate (Lenferink et al., 2024; Liu et al., 2023; Ren et al., 2021; Zhou et al., 2023). It was originally thought that bacteria carrying-out denitrification and DNRA are constrained to anoxic conditions since the two processes facilitate anaerobic respiration using NO_3_^-^ and NO_2_^-^ as terminal electron acceptors (Bonaglia et al., 2016; Caffrey et al., 2019; Jahangir et al., 2017; Yoon et al., 2015). However, microbial strains performing denitrification and DNRA in the presence of O_2_ have been recently reported, although the physiological mechanisms underlying these aerobic dissimilatory pathways remain unclear (Huang et al., 2023; Ji et al., 2015), suggesting that some microbes may simultaneously use O_2_ and inorganic N molecules as electron acceptors. The functional bacterial genes involved in denitrification and DNRA were shown to be actively transcribed under aerobic conditions and the obligate aerobic bacterium *Gemmatimonas aurantiaca*, harboring the *nosZ* gene encoding nitrous oxide reductase, was reported to significantly reduce N_2_O only when O_2_ was initially present and then depleted (Park et al., 2017). Moreover, the *nirBD* genes encoding nitrite reductase in *Pseudomonas putida* Y-9 has been described to control the DNRA process under aerobic condition (Huang et al., 2020). However, it remains unclear how these genes regulate the process at the transcript or enzymatic level. Simultaneous conversions of NO_3_^-^ to N_2_O, N_2_, and NH_4_^+^ by *Bacillus* strains under anoxic condition were also reported very recently despite the lack of genomic bases and enzymatic investigation for the bacterial strains yet (Yoon et al., 2023). However, no studies have reported the coexistence of denitrification and DNRA in a strain of *Bacillus* and *Neobacillus* under aerobic condition.

Here we report on the isolation and characterization of three bacterial isolates obtained from rice paddy field soil in Iksan City, Korea, that have the ability to carry-out aerobic denitrification and DNRA. Based on whole genome sequence analyses, strains PS2-9 and PS3-12 were identified as *Neobacillus* spp., while strain PS3-36 was *Bacillus salipaludis* and all three strains possessed the functional genes required for both denitrification and DNRA processes. They removed NO_3_^-^ efficiently with the concomitant production of NH_4_^+^ under aerobic culture conditions, and transcriptional assays showed that genes for denitrification and DNRA were simultaneously transcribed. Results of this study highlight capability of the soil facultative anaerobic bacilli strains to convert NO_3_^-^ in assimilatory and dissimilatory pathways regardless of oxygen presence, and their ecological role in terrestrial N-cycling which would be worthwhile to be investigated in future.

## Materials and Methods

### Bacterial isolation

Surface soil samples (0–2 cm depth) were collected from a rice paddy field located in Iksan city, Korea in December 2021. The soil samples were collected using a metal hand auger, placed in sterile Whirl-Pak bags, and kept on ice immediately until delivered to the lab for further processing.

For isolation of soil microbial strains utilizing NO_3_^-^ as an electron acceptor, 1 g of the soil sample was mixed with 10 ml of phosphate buffered saline (PBS, pH 7.0) and 100 µl of the 1:100 diluted soil suspension was spread onto the surface of R2A agar plates (MBcell, Korea) supplemented with 5 mM NO_3_^-^ and 10 mM acetate (R2A-NA). The plates were incubated anaerobically at 28℃ for 5 days using a GasPak EZ Anaerobe system (BD, USA). Colonies formed on the plates were re-streaked for purity onto R2A-NA agar plates to obtain well-isolated pure single colonies.

### Screening of aerobic DNRA and denitrification-performing isolates

To test NO_3_^-^ reduction and NH_4_^+^ production of the soil isolates, each of the obtained 96 pure single colonies was inoculated in 200 µl of R2A-NA broth (MBcell, Korea) contained in each well of 96-well cell culture microplate (SPL Life Sciences, Korea) and incubated for 5 days at 28℃ under aerobic or anoxic condition, which was generated using GasPak EZ container without or with GasPak EZ Anaerobe sachets, respectively.

A colorimetric plate screening method, modified from those of García-Robledo et al. (2014) and Heo et al. (2020) was used to measure NO_3_^-^, NO_2_^-^, and NH_4_^+^ in the culture supernatant (García-Robledo et al., 2014; Heo et al., 2020). The absorbances at the wavelengths described below were measured through the 96-well microplates using a Multiskan SkyHigh Microplate Spectrophotometer (Thermo Fisher Scientific, USA). Briefly, 100 µl and 5 µl supernatants of the culture in each well were transferred to their corresponding positions on two new 96-well microplates, respectively. A 95 µl aliquot of dH_2_O was added to each well of plates containing 5 µl of the culture supernatant, generating a 20-fold dilution. The wells of plates containing 100 ul of supernatant were left undiluted. For NH_4_^+^ measurement, 80 µl of NH_4_^+^ reagent A (0.2 M sodium hydroxide, 1 M sodium salicylate, and 5.88 mM sodium nitroprusside dihydrate in dH_2_O) and 20 µl of NH_4_^+^ reagent B (5.1 mM sodium dichloroisocyanurate in dH_2_O) were added to each well of the undiluted 96-well microplate. The absorbance at 660 nm was measured after 30 min incubation at 25℃, and NH_4_^+^ amount was calculated using a series of NH_4_^+^ standard concentrations at 2 mM, 1 mM, 0.75 mM, 0.5 mM, 0.25 mM, 0.1 mM, and 0.05 mM. For NO_2_^-^ measurement, 80 µl of the modified Griess reagent (Sigma-Aldrich, USA) was added to each well of the 20-fold diluted microplate, and the absorbance at 540 nm was measured after 30 min incubation at 25℃, followed by calculation of NO_2_^-^ amount using a series of NO_2_^-^ standard concentrations at 0.3 mM, 0.2 mM, 0.1 mM, 0.075 mM, 0.05 mM, 0.025 mM, and 0.01 mM. For NO_3_^-^ measurement, 20 µl of vanadium solution (1% (w/v) vanadium(III) chloride in 1 M HCl) was subsequently added to each well of the 20-fold diluted microplate after the NO_2_^-^ measurement. Absorbance was measured at 540 nm after 60 min incubation at 60℃, followed by calculation of NO_3_^-^ amount using a series of NO_3_^-^ standard concentrations at 0.3 mM, 0.2 mM, 0.1 mM, 0.075 mM, 0.05 mM, 0.025 mM, and 0.01 mM and subtraction of the NO_2_^-^ amount calculated previously. The isolates corresponding to the wells with more than 0.5 mM NH_4_^+^ and undetectable NO_3_^-^ and NO_2_^-^ under both aerobic and anoxic conditions were subjected to further analyses.

### Whole genome analyses of the soil isolates

Genomic DNA were extracted from the cell pellets of the soil isolates using the DNeasy PowerLyzer Microbial Kit (Qiagen, USA), in accordance with the manufacturer’s instruction. The eluted DNA were confirmed for the purity (1.8–2.0 at 260/280 nm and 2.0–2.2 at 260/230 nm) and concentration (>4,000 ng in 50 µl) by using a Multiskan SkyHigh Microplate Spectrophotometer with the µDrop Duo Plate (Thermo Fisher Scientific, USA). The genomic DNA samples were submitted to the KNU NGS Center (Kyungbuk National University, Daegu, Korea) for whole genome analysis by long-read sequencing by the Oxford MinION Nanopore platform (Oxford Nanopore Technologies, UK). Sequencing libraries were prepared using the Native Barcoding Kit 24 V14 and sequenced on a R10.4.1 flow cell using a MinION Mk1c device, according to the manufacturer’s manual. Base calling was done by using Guppy Basecaller v2.24 (Wick et al., 2019), and reads <1 kb and the worst 5% of read bases were filtered out by Filtlong v0.2.1 (https://github.com/rrwick/Filtlong). The quality-filtered reads were assembled de novo using Flye v2.9.1 (Kolmogorov et al., 2019).

The assembled genomes were submitted to the NCBI GenBank (under accessions numbers CP133268, CP133269, and NZ_JAVGVR000000000), and gene prediction and annotation were done by the NCBI Prokaryotic Genome Annotation Pipeline (PGAP) (Table S1) (Tatusova et al., 2016). To define species of the soil isolates, average nucleotide identity (ANI) values were calculated by the Taxonomy Check function of PGAP as comparing the assembled genomes to ones of the type strains in GenBank (Ciufo et al., 2018). Phylogenetic trees were constructed based on the 16S rRNA gene, and denitrification and DNRA functional gene sequences using maximum likelihood method with bootstrap analysis (n=1,000) of MEGA X software (Kumar et al., 2018). Pairwise blastn results between the denitrification and DNRA-functional gene clusters were visualized by using GenomeMatcher software (Ohtsubo et al., 2008).

### NO_3_^-^ conversion in the culture media with transcriptions of the DNRA and denitrification functional genes

For NO_3_^-^ conversion in a complex medium, each soil isolate was grown in R2A broth (MBcell, Korea) at 28℃ for 3 days under aerobic condition, with shaking at 100 rpm on an orbital shaker. Cells were collected from the flasks by centrifugation at 3,000 × g, were washed twice in PBS buffer (pH 7.0), and resuspended in a final volume of 300 µl. Cells were inoculated into triplicate aliquots of 10 ml R2A-NA broth, followed by incubation under aerobic in a test tube at 100 rpm on an orbital shaker, or anoxic conditions, using a Hungate anaerobic culture tube (Chemglass, USA) flushed and filled with N_2_. Cultures were incubated at 28℃ for 12 h. The aerobic conditions in the culture medium were confirmed by measuring dissolved oxygen (DO) in the culture supernatant with a portable multimeter AM70 (Apera Instrument, USA) during the growth of the soil isolates.

For NO_3_^-^ conversion in a defined minimal medium, 10 colonies of each soil isolate that were grown on R2A-NA agar plates were transferred and inoculated into 100 ml of denitrification medium (DM) (Huang et al., 2020) containing (per L) 7.00 g K2HPO_4_, 3.00 g KH_2_PO_4_, 0.10 g MgSO_4_·7H_2_O, 0.05 g FeSO_4_·7H_2_O, 0.72 g KNO_3_, and 5.13 g CH_3_COONa (pH 7.0). Triplicate cultures of each strain were incubated for 121 h at 28℃ under aerobic conditions, in Erlenmeyer flasks that were shaken at 100 rpm on an orbital shaker. Culture samples were collected at each time point and the OD_600_ of the soil isolates cultured in R2A-NA and DM was measured by using a GENESYS 30 Visible Spectrophotometer (Thermo Fisher Scientific, USA). The collected culture samples were centrifuged at 15,000 × g for 1 min, then analyzed for NO_3_^-^, NO_2_^-^, and NH_4_^+^ in the culture supernatant by using the colorimetric plate screening method described above. To estimate the fraction of NO₃⁻ reduced through assimilatory or dissimilatory pathways, each soil isolate was inoculated into 100 ml of DM medium containing 5 mM NH₄Cl instead of KNO₃. Triplicate cultures were incubated at 28°C under aerobic conditions. Culture samples were collected when the OD_600_ of the cultures approached approximately 1.00, and the NH₄⁺ concentration in the culture supernatant was analyzed.

### Transcription analyses

Total RNA was extracted from cells by using the TRIzol Max Bacterial RNA Isolation Kit (Thermo Fisher Scientific, USA), in accordance with the manufacturer’s instructions. First-strand cDNA was synthesized from the extracted RNA (100 ng) by using the DiaStar RT Kit (SolGent, Korea) with random hexamers. The oligonucleotide primer sets targeting the DNRA and denitrification functional genes were designed based on the genome sequences of the soil isolates and used to amplify *nirB*, *nrfA*, *nirK*, and *nosZ* transcripts of the strains (Table S2). Specificity of the primer sets were verified via NCBI Primer-Blast. The reaction mixture for qPCR (10 µl) contained 1X PowerUp SYBR Green Master Mix (Thermo Fisher Scientific, USA), 0.2 µM each primer, and 2 µl of cDNA samples. The real-time qPCR was performed using a QuantStudio 1 Real-Time PCR system (Thermo Fisher Scientific, USA) with the following conditions: 50°C for 120 s, and 95°C for 120 s, followed by 40 cycles of 95°C for 15 s and 60°C for 60 s. Melting curve analysis and agarose gel electrophoresis were conducted to confirm correct amplification of the PCR products. The 16S rRNA was also quantified by qPCR with Eub338 (5′-ACTCCTACGGGAGGCAGCAG-3′) and Eub518 (5′-ATTACCGCGGCTGCTGG-3′) primers to normalize the levels of the transcripts of the DNRA and denitrification functional genes (Muyzer et al., 1993).

### Nucleotide sequence accession numbers

The genome sequences obtained from this study were submitted to NCBI GenBank database (https://www.ncbi.nlm.nih.gov/genbank/) under accession numbers NZ_CP133268 (for *Neobacillus* sp. PS3-12), NZ_CP133269 (for *Neobacillus* sp. PS2-9), and NZ_JAVGVR000000000 (for *Bacillus salipaludis* PS3-36).

## Results and Discussion

### Soil microbial isolates reducing NO_3_^-^ and producing NH_4_^+^ under aerobic and anoxic conditions

Total 96 isolates capable of growing on R2A-NA agar plates under anoxic condition were obtained from the rice paddy field soil. The soil isolates were further screened for NO_3_^-^ reduction and NH_4_^+^ production by using a colorimetric plate screening method, and three soil isolates, PS2-9, PS3-12, and PS3-36, were found to reduce 5 mM NO_3_^-^ completely and produce more than 0.5 mM NH_4_^+^ under aerobic and anoxic conditions among the total isolates tested.

**Fig. 1.**
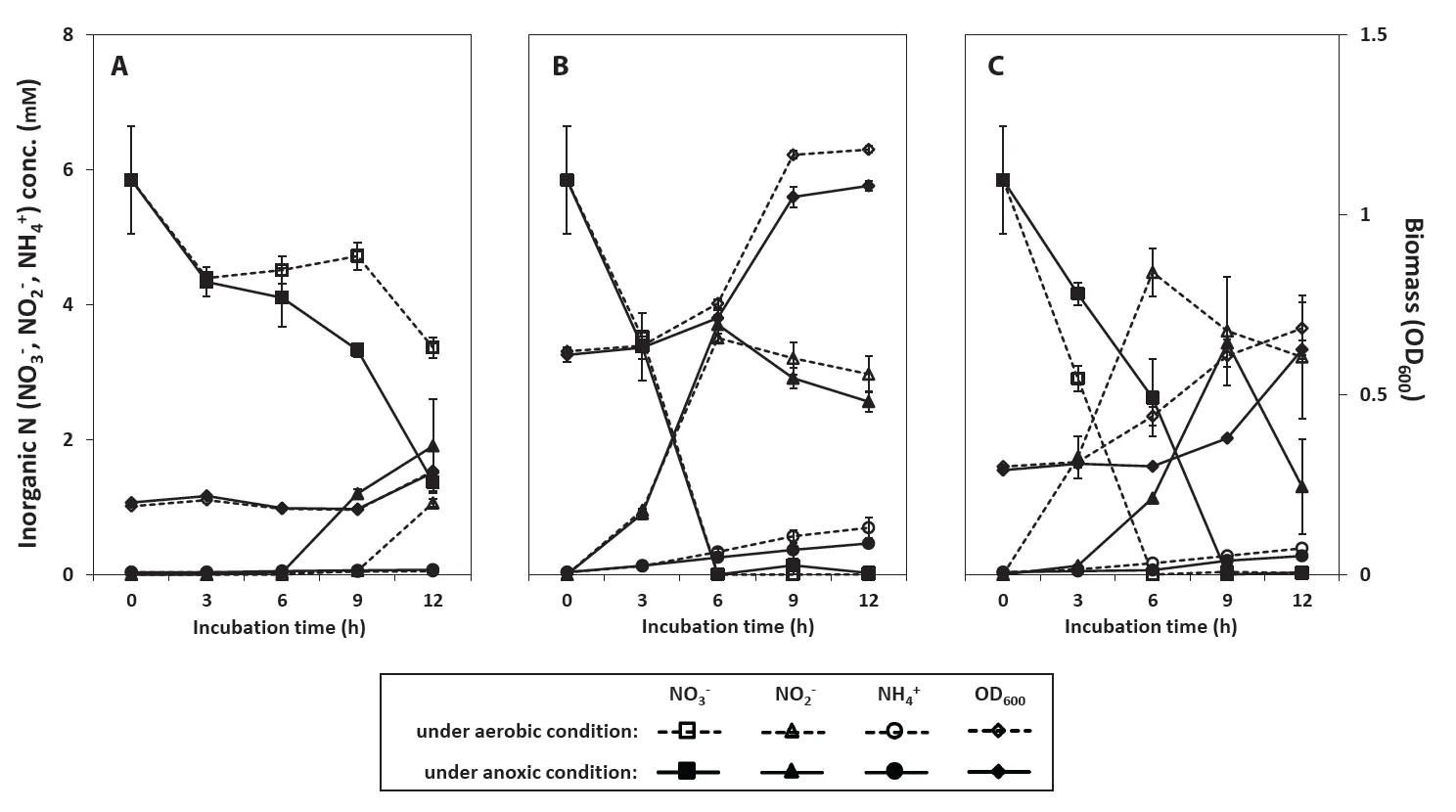
Changes in concentration of NO_3_^-^, NO_2_^-^, and NH_4_^+^ during cultivation of *Neobacillus* spp. strains PS2-9 (A) and PS3-12 (B), and *B. salipaludis* strain PS3-36 (C) in R2A-NA medium under aerobic or anoxic conditions.

Strains PS2-9, PS3-12, and PS3-36 were further assessed for their ability to reduce NO_3_^-^ and produce NH_4_^+^ in R2A-NA broth grown for 12 h under aerobic and anoxic conditions. As shown in Table S3, although DO levels decreased during cell growth in the culture medium under aerobic conditions, likely due to microbial oxygen consumption, the average DO levels of 1.89–2.96 mg/L were maintained even during the stationary phase with maximum cell density. This indicates that oxygen was continuously available to the cells throughout the aerobic incubation. As shown in Fig. 1, all of the strains were observed to grow and reduce NO_3_^-^ under both aerobic and anoxic conditions, suggesting that these soil isolates were facultative anaerobes performing NO_3_^-^ reduction regardless of the presence of oxygen. Since the quantities of reduced NO_3_^-^ were larger than the amounts of NO_2_^-^ and NH_4_^+^ produced in culture supernatants with biomass increase (Fig. 1), this suggested that NO_3_^-^ was reduced likely via both assimilatory (biomass increase) and dissimilatory (increase of extracellular NO_2_^-^ and NH_4_^+^) pathways. By estimating the NH₄⁺ required for cell growth for each strain (Table S4), the fractions of NO₃⁻ reduced via assimilatory and dissimilatory pathways were inferred (Table S5). Under aerobic conditions, approximately 85.7%, 79.3%, and 80.5% of the total reduced NO₃⁻ was processed through dissimilatory pathways by strains PS2-9, PS3-12, and PS3-36, respectively. Under anoxic conditions, these proportions increased to 93.5%, 82.6%, and 82.9% for the same strains, indicating that most of the reduced NO₃⁻ was used for dissimilatory respiration rather than biomass assimilation, with slightly higher dissimilatory activity under anoxic conditions. Since the cells can utilize other nitrogen sources (e.g., amino acids) present in the R2A-NA medium, the actual proportion of NO₃⁻ processed through dissimilatory pathways is likely higher than the estimates provided above. Strains PS3-12 and PS3-36 produced NH_4_^+^ in the supernatant after most of NO_3_^-^ was reduced with the accumulation of NO_2_^-^ (Fig. 1B & 1C). Repression of NO_2_^-^ reduction to NH_4_^+^ by NO_3_^-^ presence has been reported as the most prominent common characteristic of DNRA bacteria in several previous studies (Heo et al., 2020; Mania et al., 2016; Sun et al., 2016; Wang & Gunsalus, 2000). Since the DNRA process converts NO₃⁻ to NH₄⁺ via NO₂⁻, the decrease in NO₂⁻ concentration (1–2 mM) (Fig. 1B & 1C), followed by an increase in NH₄⁺ (around 1 mM), can be attributed to DNRA. Although strain PS2-9 also reduced NO_3_^-^ and accumulated NO_2_^-^ in the supernatant, the amount of NH_4_^+^ did not increase, possibly due to the insufficient incubation time based on its slow growth and NO_3_^-^ reduction in R2A-NA medium (Fig. 1A).

**Fig. 2.**
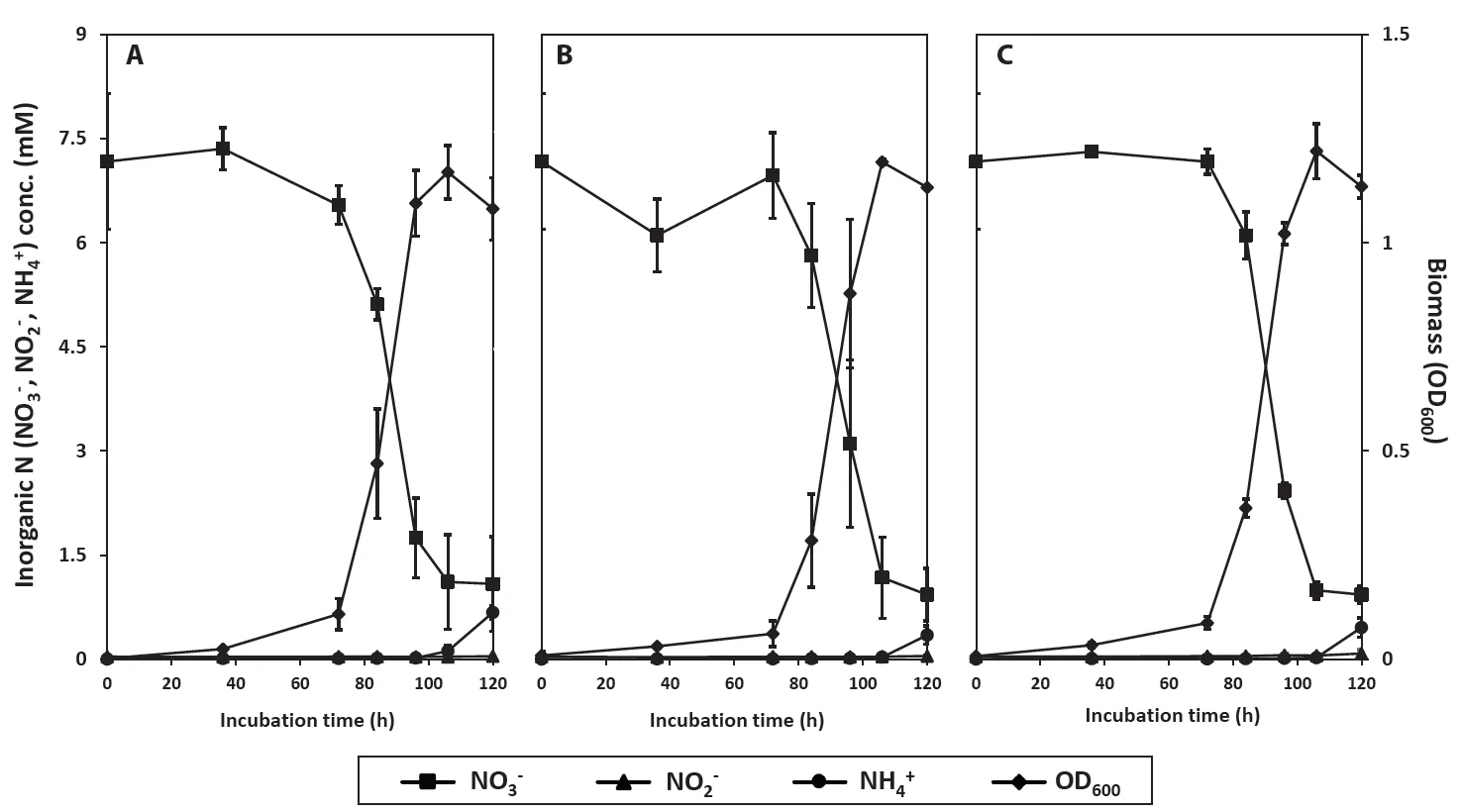
Changes in concentration of NO_3_^-^, NO_2_^-^, and NH_4_^+^ during cultivation of *Neobacillus* spp. strains PS2-9 (A) and PS3-12 (B), and *B. salipaludis* strain PS3-36 (C) in DM medium under aerobic conditions.

Since assimilatory NO_3_^-^ reduction in R2A-NA was inconclusive as the biomass increase can be done with the other available nitrogen nutrients contained in the complex medium, the aerobic conversion of NO_3_^-^ by strains PS2-9, PS3-12, and PS3-36, with an extended incubation time of 120 h, was tested in 100 ml of denitrification medium (DM) (Fig. 2). In contrast to results found using R2A-NA medium, strain PS2-9 grew faster than strains PS3-12 and PS3-36 in DM. Reduction of NO_3_^-^ was accelerated with biomass growth, and NH_4_^+^ increased in the supernatant after growth of the strains ceased. This occurred without accumulation of extracellular NO_2_^-^, indicating that a large portion of reduced NO_3_^-^ was likely assimilated into the cells during log phase of growth. Moreover, some of the NH_4_^+^ produced have possibly been released from the cells during stationary phase. Based on the NH₄⁺ required for biomass growth (Table S4), approximately 42.7%, 61.1%, and 47.2% of the total reduced NO₃⁻ was processed through dissimilatory pathways by strains PS2-9, PS3-12, and PS3-36, respectively, during cultivation in DM medium. These values are significantly lower compared to those observed during cultivation in R2A-NA medium (Wilcoxon test, P-values < 0.001). However, it should be noted that the proportion of dissimilatory pathways for NO₃⁻ reduction was estimated solely based on the observed decrease in NH₄⁺ concentrations in the culture supernatant during bacterial growth, with NH₄⁺ provided as the sole nitrogen source. Given that NH₄⁺ can be physically adsorbed onto the bacterial cell wall or temporarily stored within the cell without being assimilated, the NH₄⁺ concentration in the supernatant of cultures with high bacterial density may appear lower than the total ammonium amount minus the ammonium used for assimilation. Consequently, the actual proportion of NO₃⁻ processed through assimilatory pathways is likely lower than the estimates presented in Table S4. Taken together, strains PS2-9, PS3-12, and PS3-36 showed effective NO_3_^-^ reduction by all the three strains in complex and defined culture media under both aerobic and anoxic conditions. These strains likely performed DNRA in R2A-NA medium as they reduced NO_3_^-^ and accumulated extracellular NO_2_^-^, and then produced NH_4_^+^ when NO_3_^-^ concentration became low in the supernatant. Since R2A-NA medium contains yeast extract and partially hydrolyzed proteins as complex nitrogen source which can be readily used for cell growth, the strains likely used a portion of supplemented NO_3_^-^ for respiratory pathways, such as denitrification and DNRA. In contrast, when strains were cultured in DM under aerobic conditions, there was no detectable extracellular NO_2_^-^ or NH_4_^+^ accumulation until cell growth ceased. This suggests that the NH₄⁺ produced via NO₃⁻ reduction was predominantly utilized in assimilatory pathway. Furthermore, denitrification leading to the production of N₂O and N₂ became the dominant dissimilatory pathway, overtaking DNRA, when NO₃⁻ served as the sole electron acceptor. From a teleological point of view, it makes sense that NO_3_^-^ is mostly used for biomass increase when molecular O_2_ is present and used as a respiratory electron acceptor for these facultative anaerobes when NO_3_^-^ is the sole nitrogen source. Moreover, NH_4_^+^ from respiratory ammonification would be released when the cells do not require much of NO_3_^-^ for growth. Several denitrifying bacteria have been reported to transform NO_3_^-^ to gaseous and biomass N, and the ratio between respired and assimilated NO_3_^-^ have been shown to vary depending on the studies conducted with different bacterial species and culture conditions (Yang et al., 2021; Yao et al., 2013; Zhang et al., 2018). Our strains appear to regulate assimilatory and dissimilatory NO_3_^-^ reduction pathways according to their growth condition such as availability of nitrogen source other than NO_3_^-^.

### Strain identification

**Fig. 3.**
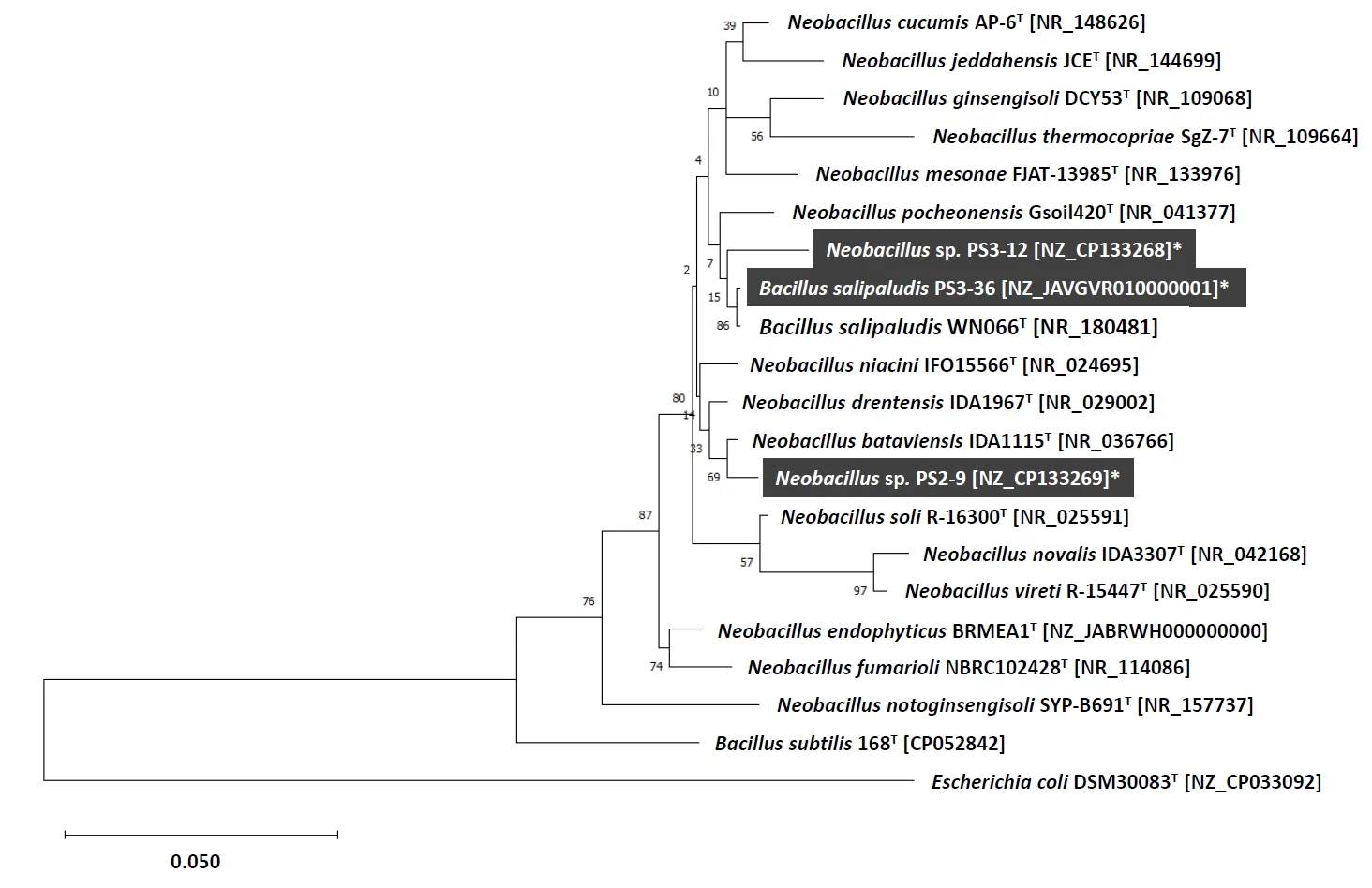
Phylogenetic tree constructed based on 16S rRNA gene sequences by using the maximum likelihood method (bootstrap values (%) were generated from 1,000 replicates). GenBank accession numbers are shown in square brackets, and strains highlighted in dark gray were obtained in this study.

In order to taxonomically identify the isolates and examine the functional genes involved in denitrification and DNRA, we sequenced the complete genomes of strains PS2-9, PS3-12, and PS3-36 using the Oxford MinION Nanopore platform. The resulting high-quality sequences were assembled to total lengths of 5.2 to 6.7 Mbps per genome, with >254x coverage (Table S1). The strain PS3-36 was identified as *Bacillus salipaludis*, based on an average nucleotide identity (ANI) value of >99% identity with >93% query coverage with *B. salipaludis* WN066T (GenBank accession NZ_SMYO00000000) and 16S rRNA gene sequences in GenBank. Although strains PS2-9 and PS3-12 were identified to be members of genus *Neobacillus*, based on 16S rRNA gene sequences, their ANI values to each other and known type strains of *Neobacillus* in GenBank were below species cutoff value (>95% identity with >60% query coverage), indicating that PS2-9 and PS3-12 are likely novel *Neobacillus* species strains. Interestingly, *B. salipaludis*, including strain PS3-36, is more closely related with *Neobacillus* spp. than *Bacillus subtilis* (the type species of *Bacillus*) based on 16S rRNA phylogeny (Fig. 3).

The genus *Bacillus* is comprised of diverse bacterial members that often show insufficient divergence in 16S rRNA genes to identify strains to the species level. Consequently, traditional analysis of 16S rRNA nucleotide sequence data does not provide sufficient resolution at the species level for these bacteria (Maughan & Van der Auwera, 2011). This indicates that 16S rRNA phylogenetic tree would not provide clear differentiation between existing and newly classified *Bacillus* species as shown in Fig. 3. However, based on complete genome sequencing, ANI and query coverage, strain PS3-36 was identified as *B. salipaludis* which is a novel soil *Bacillus* species introduced recently and unknown for its nitrogen cycling functions (Xue et al., 2021). In contrast, strains PS2-9 and PS3-12 were assumed to be novel *Neobacillus* species since they had no match with known genomes of type strains satisfying the required ANI cutoff value for species identification. Further study on the strains would be continued to report novel bacilli strains which may play important roles in soil nitrogen cycle.

### Elucidation of functional genes involved in denitrification and DNRA

**Fig. 4.**
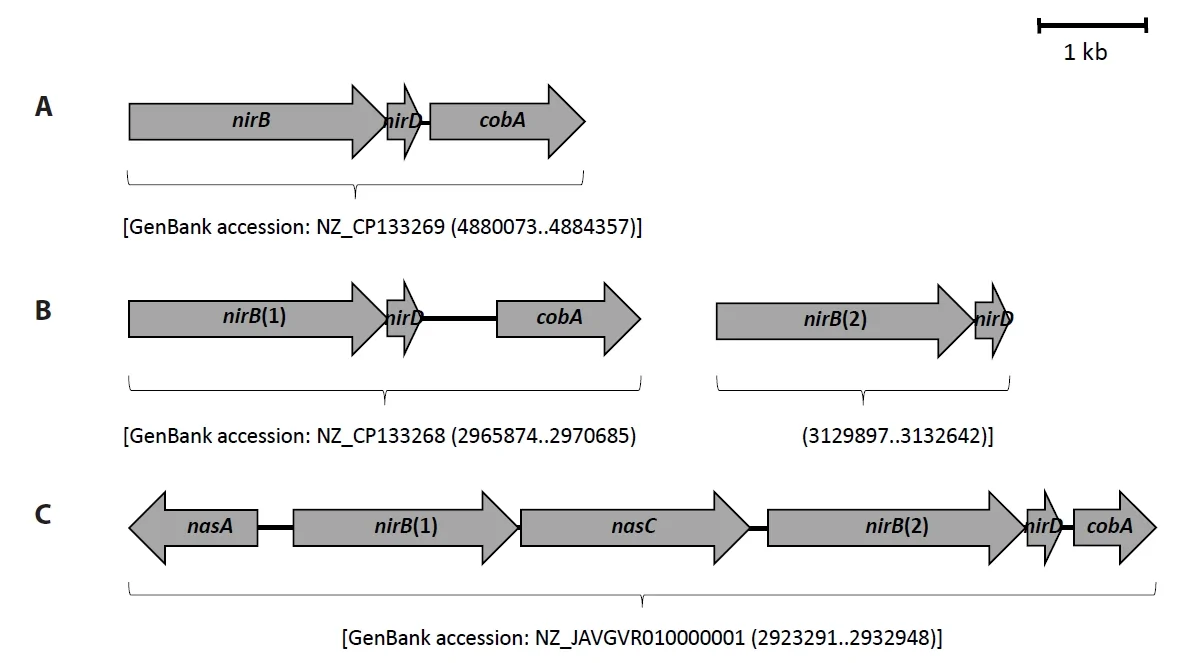
Clusters of *nirB* genes identified on genomes of *Neobacillus* spp. strains PS2-9 (A) and PS3-12 (B), and *B. salipaludis* strain PS3-36 (C).

As shown in Table 1, denitrification and DNRA functional genes were identified in genomes of strains PS2-9, PS3-12, and PS3-36. Moreover, while the DNRA functional genes *nirB* and *nrfA* were found in the genomes of the *Neobacillus* strains PS2-9 and PS3-12, only *nirB* was identified in the genome of the *B. salipaludis* strain PS3-36. Interestingly, strains PS3-12 and PS3-36 possess two *nirB* genes (arbitrarily named as *nirB*(1) and *nirB*(2)) and nucleotide sequence similarities between those *nirB* genes are below 70% (Table 2), indicating that the *nirB* genes within a single strain would have originated from different sources, and it is presumed that one or both *nirB* genes were acquired through horizontal gene transfer (Marzocchi et al., 2022). While the *nirB* gene in strains PS2-9 and PS3-12 was observed to be present with *nirD*, the *nirB*(1) gene of strain PS3-36 was surrounded by assimilatory nitrate reductase (Nas)-encoding genes and followed by *nirB*(2) and *nirD* (Fig. 4). The gene *cobA* was observed in most of *nirBD* gene clusters identified in this study, except for the *nirB*(2)-*nirD* cluster present in strain PS3-12.

Taken together, all of the strains possess denitrification and DNRA functional genes, indicating that these bacilli strains have the genotypic potential to reduce NO_3_^-^ to N_2_ gas as well as NH_4_^+^. The nitrogen transforming gene cluster found in *B. salipaludis* strain PS3-36 contained *nasA*, *nirB*(1), *nasC*, *nirB*(2), *nirD*, and *cobA* which somewhat resembles the *nasB* operon and *nasA* required for NO_3_^-^/NO_2_^-^ assimilation in *Bacillus subtilis* (Ogawa et al., 1995). Further studies are needed to elucidate whether the *nirB* gene cluster of strain PS3-36 is a common genetic feature among other members of the genus *Bacillus*, and to determine how the genes function for NO_3_^-^ assimilation and DNRA.

### Cotranscription of denitrification and DNRA functional genes

Total RNA was extracted from the cells of strains PS2-9, PS3-12, and PS3-36 grown in DM medium under aerobic conditions for various times. The first-strands of cDNA synthesized from the collected RNA samples and genomic DNA extracted from *Neobacillus* strains PS2-9 and PS3-12 and *B. salipaludis* strain PS3-36 were used as templates for real-time qPCR (RTqPCR) amplifying 16S rRNA, *nirK*, *nosZ*, *nirB*, and *nrfA*.

**Fig. 5.**
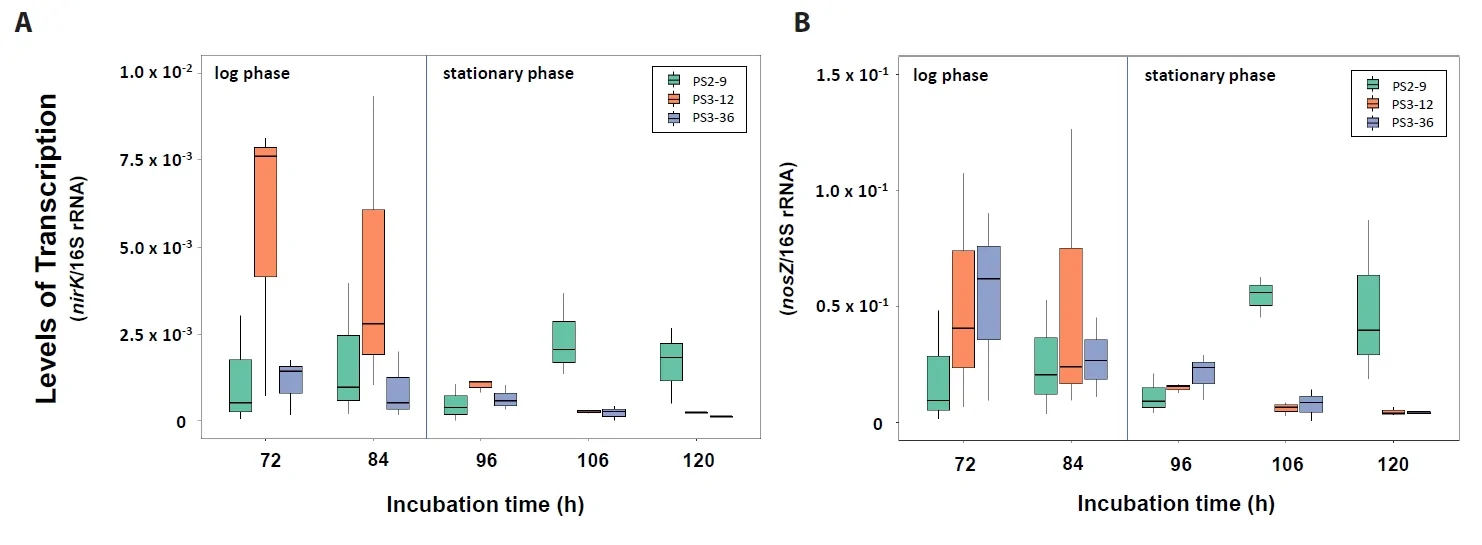
Transcription levels of *nirB* genes in *Neobacillus* spp. strains PS2-9 (A) and PS3-12 (B), and *B. salipaludis* strain PS3-36 (C), and *nrfA* genes of strains PS2-9 (D) and PS3-12 (E) during cultivation in DM medium under aerobic conditions. Transcription levels were normalized by amount of 16S rRNA.

As shown in Fig. 5, the transcription levels of the denitrification functional genes *nirK* and *nosZ* in *Neobacillus* strains PS3-12 and *B. salipaludis* strain PS3-36 were significantly greater at 72 and 84 h compared to those from cells collected at 96, 106, and 120 h (Wilcoxon test, P-values < 0.05). In contrast, *Neobacillus* strain PS2-9 had significantly higher transcription levels of *nirK* and *nosZ* at 106 and 120 h, than ones at 72, 84, 96 h (Wilcoxon test, P-values < 0.05). Consequently, while strains PS3-12 and PS3-36 would likely carry-out denitrification during the log phase of growth when they started to reduce NO_3_^-^ exponentially, denitrification by strain PS2-9 would be more active at later log phase and stationary phases when the strain reached maximum biomass (Fig. 2).

**Fig. 6.**
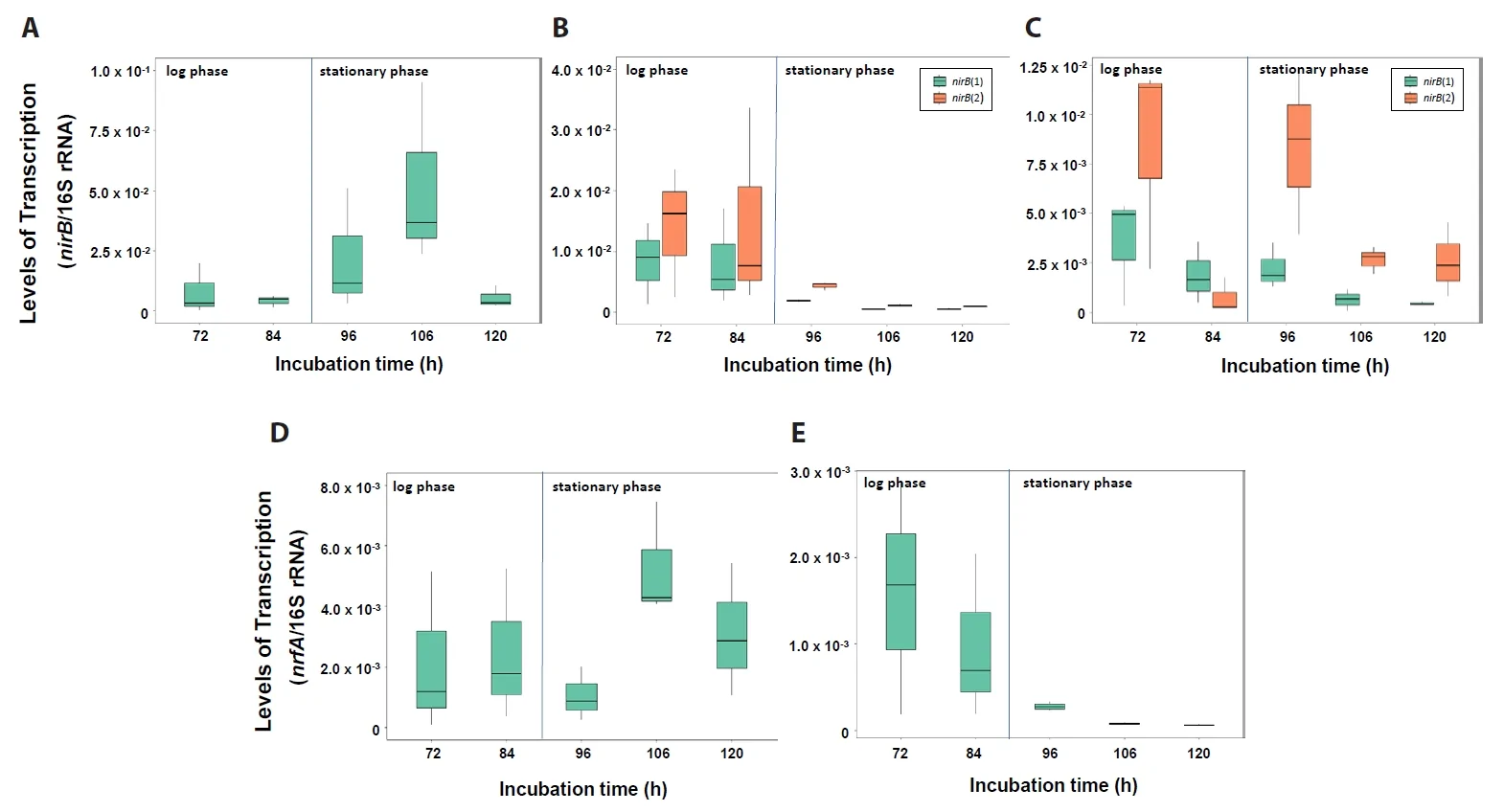
Transcription levels of *nirK* (A) and *nosZ* (B) genes in strains *Neobacillus* spp. strains PS2-9 and PS3-12, and *B. salipaludis* strain PS3-36 during cultivation in DM medium under aerobic condition. Transcription levels were normalized by amount of 16S rRNA.

Transcription levels of the DNRA functional genes *nirB* and *nrfA* are shown in Fig. 6. The *Neobacillus* strain PS2-9 showed greatest transcription levels of *nirB* and *nrfA* at 106 h (Fig. 6A & 6D). Significantly higher transcriptional levels of *nirB* (1), *nirB* (2), and *nrfA* genes in *Neobacillus* strains PS3-12 were seen at 72 and 84 h of growth, compared to those at 96, 106, and 120 h (Wilcoxon test, P-values < 0.001) (Fig. 6B & 6E). Similarly, *B. salipaludis* strain PS3-36 was found to transcribe significantly more *nirB* (1) at 72, 84, and 96 h, compared to levels seen at 106 and 120 h (Wilcoxon test, P-values < 0.01) (Fig. 6C). Moreover, transcription levels of *nirB* (2) in *B. salipaludis* strain PS3-36 was significantly higher at 72 and 96 h than that seen at 106 and 120 h (Fig. 6C). In short, nitrate ammonification conducted by *nirB* and/or *nrfA* would be more active at stationary growth phase of strain PS2-9 and log growth phase of strains PS3-12 and PS3-36, suggesting that each strain would have their own regulation for the DNRA gene expression. Interestingly, the two distantly related *nirB* genes with < 70% nucleotide sequence similarities were transcribed simultaneously at significantly different transcription levels in strains PS3-12 and PS3-36 when reducing NO_3_^-^ without production of extracellular NH_4_^+^ (Wilcoxon test, P-values < 0.05) (Fig. 6B & 6C). This strongly suggests that two different *nirB* genes likely work together for NO_3_^-^ assimilation. And likely under different regulatory control.

Taken together, although transcription levels of denitrification and DNRA functional genes in strains PS2-9, PS3-12, and PS3-36 were observed to vary according to incubation time, NO_3_^-^ reduction would be contributed by both denitrification and nitrate ammonification as those functional genes were cotranscribed during cultivation of the strains under aerobic condition. This possibly indicates that these bacterial strains reduce NO_3_^-^ to N_2_ and NH_4_^+^ simultaneously. The DNRA functional gene *nrfA* encodes a periplasmic nitrite reductase reducing NO_2_^-^ to NH_4_^+^ and is used as a molecular marker for DNRA activity (Pandey et al., 2020). Nevertheless, in this study, extracellular NH_4_^+^ did not accumulate in the culture medium of *Neobacillus* strains PS2-9 and PS3-12 even with the *nrfA* gene transcription during incubation in DM under aerobic conditions. This suggested that *nrfA* likely also functions in the assimilatory pathway, like *nirB*, which has been reported to perform both assimilatory and dissmilatory NO_2_^-^ reduction to NH_4_^+^ (Pandey et al., 2020). Most of the NH_4_^+^ produced by both NirB and NrfA in cytoplasm and periplasm, respectively, were likely assimilated into bacterial biomass under growth condition with NO_3_^-^ as a sole nitrogen source. Nevertheless, it must be noted that while transcript analysis provides valuable insights into gene expression levels and important data on the potential for a process to occur, it does not always provide sufficient evidence to confirm that the corresponding processes are actually taking place within the cell. The interpretation of the transcript analysis results in this study should be validated with enzymatic and metabolic analyses in future study.

## Conclusion

In this study, the soil bacteria *Neobacillus* spp. strains PS2-9 and PS3-12, and *B. salipaludis* strain PS3-36 were shown to be capable of carrying-out assimilatory and dissimilatory NO_3_^-^ reduction under aerobic and anaerobic conditions. This is a novel report describing the coexistence of denitrification, DNRA, and assimilatory NO_3_^-^ reduction in soil bacilli strains under aerobic and anoxic conditions, and the strains are valuable microbial resource to be studied for regulation of their nitrogen metabolism and contribution to the soil nitrogen cycle. While most existing studies on the regulation and differences between soil denitrification and DNRA have focused on the entire soil ecosystem including total microbial community without ecophysiology of N cycling microbes, further detailed studies on these strains with isotope tracing techniques would enhance our understanding of terrestrial microbial N-cycling as they may play a pivotal role in the soil microbial community.

## Figures and Tables

**Table 1.** Locus tags of denitrification and DNRA functional genes in the GenBank files^a^ of the genomes of *Neobacillus* spp. strains PS2-9 and PS3-12, and *B. salipaludis* strain PS3-36

N cycle functional genes	Strain ID			
	PS2-9	PS3-12	PS3-36	
*narG*	RCG25_RS10395	RCG17_RS03300	RCG21_RS14720	
*nirK*	RCG25_RS04160	RCG17_RS24495	RCG21_RS29420	
*norB*	RCG25_RS09500	RCG17_RS03055	RCG21_RS24235	
*nosZ*	RCG25_RS00605	RCG17_RS12615	RCG21_RS02000	
*nirB* (1)	RCG25_RS24395	RCG17_RS15170	RCG21_RS14475	
*nirB* (2)	NI^2^	RCG17_RS15955	RCG21_RS14485	
*nrfA*	RCG25_RS06415	RCG17_RS10725	NI^b^	

a GenBank files NZ_CP133268 and NZ_CP133269 for *Neobacillus* sp. PS3-12 and PS2-9, respectively, and NZ_JAVGVR000000000 for *B. salipaludis* strain PS3-36.

b NI: not identified

**Table 2.** Blastn-based sequence similarities (%) between *nirB* genes identified in the genomes of *Neobacillus* spp. strains PS2-9 and PS3-12, and *B. salipaludis* strain PS3-36

*nirB* genes	PS2-9 *nirB*	PS3-12 *nirB* (1)	PS3-12 *nirB* (2)	PS3-36 *nirB* (1)	PS3-36 *nirB* (2)
PS2-9 *nirB*	100	76.13 (^*^Q 99)	65.95 (Q 92)	64.13 (Q 40)	69.16 (Q 82)
PS3-12 *nirB* (1)	76.13 (^*^Q 99)	100	67.54 (Q 97)	64.85 (Q 40)	65.99 (Q 92)
PS3-12 *nirB* (2)	65.95 (Q 92)	67.54 (Q 97)	100	67.01 (Q 45)	69.47 (Q 91)
PS3-36 *nirB* (1)	64.13 (Q 40)	64.85 (Q 40)	67.01 (Q 45)	100	66.26 (Q 48)
PS3-36 *nirB* (2)	69.16 (Q 82)	65.99 (Q 92)	69.47 (Q 91)	66.26 (Q 48)	100

* Q: query % coverage

## Supplementary Information

Table S1. Summary of genome sequences of *Neobacillus* spp. PS2-9 and PS3-12, and *Bacillus salipaludis* PS3-36 obtained by using Oxford Nanopore MinION sequencing platform

Table S2. The oligonucleotide primer sets used for quantitative real-time PCR amplifying denitrification and DNRA functional genes of the soil strains PS2-9, PS3-12, and PS3-36

Table S3. Dissolved oxygen (DO) levels (mg/L) in the R2A-NA culture medium during the growth of soil strains PS2-9, PS3-12, and PS3-36 incubated under aerobic conditions at 28°C on an orbital shaker set to 100 rpm

Table S4. Decreased NH₄⁺ concentrations in DM medium supplemented with NH₄⁺ as the sole nitrogen source during the cultivation of *Neobacillus* spp. strains PS2-9 and PS3-12, and *B. salipaludis* strain PS3-36, as they approached an OD_600_ of 1.00

Table S5. Estimated NO_3_^-^ reduced via assimilatory and dissimilatory pathways after cultivation of *Neobacillus* spp. strains PS2-9 and PS3-12, and *B. salipaludis* strain PS3-36 in R2A-NA medium for 12 h under aerobic and anoxic conditions (Fig. 1)

Table S6. Estimated NO_3_^-^ reduced via assimilatory and dissimilatory pathways after cultivation of *Neobacillus* spp. strains PS2-9 and PS3-12, and *B. salipaludis* strain PS3-36 in DM medium for 120 h under aerobic conditions (Fig. 2)
