# Supplementary material for: Dissimilatory nitrate reductions in soil Neobacillus and Bacillus strains under aerobic condition
Source: J Microbiol. 2025 Feb 27;63(2):e2411019. doi: 10.71150/jm.2411019 (PMC13577133; doi:10.71150/jm.2411019)
Supplement: Supplementary file 1 [file jm-2411019-Supplementary-Table-S1.pdf]

Table S1. Summary of genome sequences of *Neobacillus* spp. PS2-9 and PS3-12, and *Bacillus salipaludis* PS3-36 obtained by using Oxford Nanopore MinION sequencing platform

Strains	*Neobacillus* sp. PS2-9	*Neobacillus* sp. PS3-12	*Bacillus salipaludis* PS3-36
Total output bases	1.48 Gb	1.42 Gb	2.26 Gb
Number of contigs	1	1	2
Length of 1st contig (bp)	5,192,194	5,612,023	6,261,405
Length of 2nd contig (bp)	-	-	477,182
Contig form	Circular	Circular	Linear
Total length of genome (bp)	5,192,194	5,612,023	6,738,587
Genome coverage (fold)	285	254	337
CDS (with protein)	5,205	5,453	6,442
CDS (without protein)	60	315	202
tRNAs	125	123	176
rRNAs	45	51	57
GenBank accession No.	NZ_CP133269	NZ_CP133268	NZ_JAVGVR000000000
