# Supplementary material for: Dissimilatory nitrate reductions in soil Neobacillus and Bacillus strains under aerobic condition
Source: J Microbiol. 2025 Feb 27;63(2):e2411019. doi: 10.71150/jm.2411019 (PMC13577133; doi:10.71150/jm.2411019)
Supplement: Supplementary file 2 [file jm-2411019-Supplementary-Table-S2.pdf]

Table S2. The oligonucleotide primer sets used for quantitative real-time PCR amplifying denitrification and DNRA functional genes of the soil strains PS2-9, PS3-12, and PS3-36

Primer name	Nucleotide sequence	Target gene	Target strain	Expected amplicon size (bp)
Eub_338	ACTCCTACGGGAGGCAGCAG	16 rRNA	bacteria	197
Eub_518	ATTACCGCGGCTGCTGG			
PSY_nosZ_F	CYGGMGGTCACTCTGGACAA	*nosZ*	PS2-9, PS3-12, PS3-36	315
PSY_nosZ_R	ACRAABGCKGCACAGTGMGG			
PS2-9_nirK_F	TTGCTAGCAGCGGTTTGTCA	*nirK*	PS2-9	296
PS2-9_nirK_R	AGCCGATAATCCAGGGGTGT			
PS3-12_nirK_F	CATTTAACGGACAAGCGCCG		PS3-12	200
PS3-12_nirK_R	CACTCCCGGATTGTCAGCAG			
PS3-36_nirK_F	TCGTAGCGAAAGTTGGCGAT		PS3-36	173
PS3-36_nirK_R	TGCTCCACCACTAGCAGGAA			
PS2-9_nirB_F	CTGGAAACGGTGGGGTAGAC	*nirB*	PS2-9	136
PS2-9_nirB_R	TTAGACGTCCGCTCCAGGTA			
PS3-12_nirB(1)_F	TGCACTACTTTAAGCCGCGA	*nirB*(1)	PS3-12	130
PS3-12_nirB(1)_R	TAAGAGCAGGGCGGCATTTT			
PS3-12_nirB(2)_F	GGGACMTAYTCYGTTGTMCCR	*nirB*(2)		185
PS3-12_nirB(2)_R	GGCATDTCRAGMTCBKCCC			
PS3-36_nirB(1)_F	TCGGGAACAGCCGAGTAGA	*nirB*(1)	PS3-36	146
PS3-36_nirB(1)_R	ATTGACAAGAATCGCCCGGT			
PS3-36_nirB(2)_F	GGTTGGAGTTCGGAGGAAGG	*nirB*(2)		130
PS3-36_nirB(2)_R	TATTGGCATGCATCCGCTCA			
ASH_nrfA_F	GGCTGYTCYGAYTGCCATGACC	*nrfA*	PS2-9, PS3-12	193
ASH_nrfA_R	TCCAWGGGAAGGTKACTTCRCT			
