# Supplementary material for: Dissimilatory nitrate reductions in soil Neobacillus and Bacillus strains under aerobic condition
Source: J Microbiol. 2025 Feb 27;63(2):e2411019. doi: 10.71150/jm.2411019 (PMC13577133; doi:10.71150/jm.2411019)
Supplement: Supplementary file 3 [file jm-2411019-Supplementary-Table-S3.pdf]

Table S3. Dissolved oxygen (DO) levels (mg/L) in the R2A-NA culture medium during the growth of soil strains PS2-9, PS3-12, and PS3-36 incubated under aerobic conditions at 28°C on an orbital shaker set to 100 rpm

Incubation time (h)	PS2-9		PS3-12		PS3-36	
	OD_{600}	DO	OD_{600}	DO	OD_{600}	DO
0	0.06 ± 0.02	8.45 ± 0.13	0.05 ± 0.01	8.45 ± 0.13	0.05 ± 0.00	8.45 ± 0.13
10	0.12 ± 0.08	4.78 ± 1.33	0.21 ± 0.13	4.22 ± 2.01	0.22 ± 0.18	4.02 ± 1.87
20	1.07 ± 0.03	1.89 ± 0.62	1.14 ± 0.06	2.96 ± 0.64	1.09 ± 0.10	2.54 ± 0.42
25	1.11 ± 0.03	2.32 ± 0.42	1.11 ± 0.01	2.51 ± 0.72	1.03 ± 0.08	2.97 ± 0.28
