# Supplementary material for: Dissimilatory nitrate reductions in soil Neobacillus and Bacillus strains under aerobic condition
Source: J Microbiol. 2025 Feb 27;63(2):e2411019. doi: 10.71150/jm.2411019 (PMC13577133; doi:10.71150/jm.2411019)
Supplement: Supplementary file 4 [file jm-2411019-Supplementary-Table-S4.pdf]

Table S4. Decreased NH_4^+ concentrations in DM medium supplemented with NH_4^+ as the sole nitrogen source during the cultivation of *Neobacillus* spp. strains PS2-9 and PS3-12, and *B. salipaludis* strain PS3-36, as they approached an OD_{600} of 1.00

Strain ID	Required NH_4^+ for growth (mM/OD_{600})
PS2-9	3.23 ± 0.12
PS3-12	2.16 ± 0.23
PS3-36	2.92 ± 0.07
