# Supplementary material for: Dissimilatory nitrate reductions in soil Neobacillus and Bacillus strains under aerobic condition
Source: J Microbiol. 2025 Feb 27;63(2):e2411019. doi: 10.71150/jm.2411019 (PMC13577133; doi:10.71150/jm.2411019)
Supplement: Supplementary file 5 [file jm-2411019-Supplementary-Table-S5.pdf]

Table S5. Estimated NO_3^- reduced via assimilatory and dissimilatory pathways after cultivation of *Neobacillus* spp. strains PS2-9 and PS3-12, and *B. salipaludis* strain PS3-36 in R2A-NA medium for 12 h under aerobic and anoxic conditions (Fig. 1)

Strain ID	Aerobic			Anaerobic		
	OD_{600} increase	NO_3^- reduced in assimilatory pathway (mM)	NO_3^- reduced in dissimilatory pathway (mM)	OD_{600} increase	NO_3^- reduced in assimilatory pathway (mM)	NO_3^- reduced in dissimilatory pathway (mM)
PS2-9	0.11 ± 0.01	0.35 ± 0.01	2.13 ± 0.02	0.09 ± 0.00	0.29 ± 0.01	4.19 ± 0.05
PS3-12	0.56 ± 0.01	1.21 ± 0.13	4.63 ± 0.24	0.47 ± 0.01	1.01 ± 0.11	4.81 ± 0.31
PS3-36	0.38 ± 0.07	1.11 ± 0.03	4.71 ± 0.17	0.34 ± 0.03	0.99 ± 0.02	4.82 ± 0.23
