# Supplementary material for: Dissimilatory nitrate reductions in soil Neobacillus and Bacillus strains under aerobic condition
Source: J Microbiol. 2025 Feb 27;63(2):e2411019. doi: 10.71150/jm.2411019 (PMC13577133; doi:10.71150/jm.2411019)
Supplement: Supplementary file 6 [file jm-2411019-Supplementary-Table-S6.pdf]

Table S6. Estimated NO_3^- reduced via assimilatory and dissimilatory pathways after cultivation of *Neobacillus* spp. strains PS2-9 and PS3-12, and *B. salipaludis* strain PS3-36 in DM medium for 120 h under aerobic conditions (Fig. 2)

Strain ID	OD_{600} increase	NO_3^- reduced in assimilatory pathway (mM)	NO_3^- reduced in dissimilatory pathway (mM)
PS2-9	1.08 ± 0.07	3.49 ± 0.08	2.59 ± 0.07
PS3-12	1.12 ± 0.01	2.43 ± 0.14	3.81 ± 0.12
PS3-36	1.13 ± 0.03	3.29 ± 0.11	2.94 ± 0.09
